# Intraindividual comparison of imaging modalities in anterolateral thigh perforator mapping

**DOI:** 10.1038/s41598-026-64164-w

**Published:** 2026-08-12

**Authors:** Alexander Geierlehner, Raymund E. Horch, Michael Uder, Matthias May, Andreas Arkudas, Wibke Müller-Seubert, Maximilian C. Stumpfe, Marco Wiesmueller, Aijia Cai

**Affiliations:** 1 Department of Plastic and Hand Surgery, Laboratory for Tissue Engineering and Regenerative Medicine, University Hospital Erlangen, Friedrich-Alexander-University Erlangen-Nürnberg (FAU), Erlangen, Germany; 2 Institute of Radiology, University Hospital Erlangen, Friedrich-Alexander-University Erlangen-Nürnberg (FAU), 91054 Erlangen, Germany

**Keywords:** Perforator vessel mapping, Imaging modality, Anterolateral thigh flap, Reconstructive surgery, Anatomy, Diseases, Health care, Medical research

## Abstract

Accurate knowledge of perforator vessels is crucial for successful perforator flap surgery. The purpose of this prospective, single-center study was to compare three imaging modalities, color-coded duplex sonography (CCDS), flat-panel computed tomography (FPCT) and non-contrast time-of-flight magnetic resonance angiography (TOF-MRA), for preoperative perforator mapping in anterolateral thigh (ALT) free flap reconstruction. Assessment of detection rates, accuracy of the fascial emergence point localization and the accuracy in predicting the subfascial course of perforators was performed using intraoperative findings as the reference standard. This study included ten patients, with 17 perforators identified preoperatively and 15 included in the ALT flap design. CCDS demonstrated the highest observed sensitivity for perforator detection (94%), followed by TOF-MRA (91%) and FPCT (87%) (*p* = 0.82). FPCT showed a slightly higher proportion (25%) of markings beyond a 2 cm radius from the fascial emergence point compared to CCDS (6%) and TOF-MRA (0%) (*p* = 0.31). One FPCT inaccuracy was linked to mobilization of the thigh during the examination. Regarding prediction of the perforator course, FPCT showed lower observed accuracy (50%) compared with CCDS (93%) and TOF-MRA (82%) with an overall statistically significant difference between modalities (*p* = 0.033). This study suggests that CCDS provides high accuracy for perforator detection and prediction of the perforator course, however requiring a certain amount of examiner experience. TOF-MRA, a non-contrast angiographic imaging method, shows to be a reliable, less-examiner dependent alternative making it especially suitable for centers with lower surgical volumes. FPCT is particularly valuable in patients who already require contrast-enhanced vascular imaging of the affected extremity. However, accurate assessment of perforators using FPCT requires consistent immobilization of the patient’s lower extremity.

**Supplementary Information:**

The online version contains supplementary material available at 10.1038/s41598-026-64164-w.

## Introduction

The anterolateral thigh (ALT) flap, first introduced by Song et al. in 1984, was originally described as a septocutaneous flap based on perforators traversing the intermuscular septum^1^. Subsequent anatomical investigations have demonstrated that its vascular supply may derive from perforators following intramuscular, septal or mixed courses originating from the descending or the oblique branch of the lateral circumflex femoral artery^2,3^. With a comprehensive understanding of the perforator vascular territory (“perforasome” concept)^4–8^, the ALT flap has evolved into a principal tool in modern reconstructive surgery^9–13^. However, its highly variable vascular anatomy presents a major challenge. Up to 80% of perforators follow an intramuscular course^14^ and in about 5.5% they may be absent entirely^15^. This variability increases operative complexity, complication rates and healthcare costs^16^. Precise knowledge of perforator number, caliber, fascial emergence point and course is therefore crucial for optimizing flap design and surgical outcomes^17,18^. Preoperative perforator mapping has been shown to significantly reduce operative time and complication rates^19–21^. Several imaging modalities including hand-held Doppler, color-coded duplex sonography (CCDS), computed tomography angiography (CTA) and digital subtraction angiography (DSA) have been evaluated with varying accuracy and feasibility^17,22–26^. Flat-panel computed tomography (FPCT) enables CT-like imaging within the angiography suite offering high spatial resolution but requiring contrast medium, radiation exposure and arterial access^27^. By contrast, Time-of-Flight Magnetic Resonance Angiography (TOF-MRA) provides high-resolution contrast- and radiation-free vascular imaging, in particular this technique highly benefits from the inherent superior soft tissue contrast compared to X-Ray based modalities^28^. This prospective study aims to evaluate and compare CCDS, FPCT and TOF-MRA for ALT perforator mapping, with particular emphasis on visualizing the perforator course and the fascial emergence point.

## Materials and methods

### Patient population

This prospective single-center study was approved by the Institutional Ethics Committee (registration number: 23-275-B) and conducted in accordance with the Declaration of Helsinki. Patients requiring reconstructive surgery with an ALT free flap and undergoing preoperative CCDS, FPCT and TOF-MRA of the thigh between May 2023 and May 2025 were eligible for inclusion. Written informed consent was obtained from all participants. The manuscript was checked against the Strengthening the Reporting of Observational Studies in Epidemiology (STROBE) checklist (Online Resource 1). Only patients with extremity defects were included, as FPCT of the donor thigh is routinely used as part of digital subtraction angiography at our clinic prior to free flap surgery. It is therefore clinically indicated for preoperative perforator mapping and was not performed specifically for the purpose of this study. Exclusion criteria comprised contraindications to MRA (pregnancy, presence of pacemaker, metal fragments, unsuitable implants and claustrophobia) and FPCT (hyperthyroidism, nephropathy grade of 4 or higher and a history of iodine intolerance). No specific contraindications applied to CCDS.

### Color-coded duplex sonography (CCDS)

**Figure 1:**
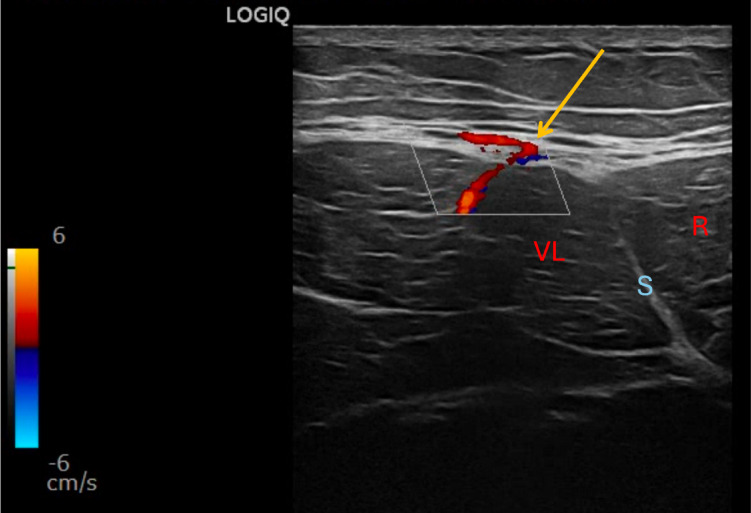
Color-Coded Duplex Sonography of a transverse Color-Flow picture cut of a right thigh which illustrates the intramuscular course of a perforator with its emergence point (orange arrow) at the deep fascia. Abbreviation: VL: vastus lateralis muscle; S = septum between the rectus femoris and the vastus lateralis muscle; R = rectus femoris muscle.

CCDS was performed with the LOGIQ E10s ultrasound (GE Healthcare Technologies Inc., Chicago, Illinois, USA) using a linear transducer (ML4-20 MHz). A standardized examination procedure with the patient placed in supine position and markings of the intermuscular septum (IS) line, between rectus femoris (RF) and vastus lateralis (VL) muscle from the anterior superior iliac spine (ASIS) to patella’s superolateral border and its midpoint were performed at the donor thigh^29^. Depending on the location of the defect, the contralateral thigh was chosen as donor site. The identified and selected perforators were traced to the surface, starting from the descending branch of the lateral circumflex femoral artery, allowing the determination of the perforator course (septal/muscular) (Fig. 1). The fascial emergence point was marked perpendicular at the skin surface. All examinations were performed by the same examiner prior to FPCT and TOF-MRA in order to avoid confirmation or anchoring bias.

### Flat-panel computed tomography (FPCT)

**Figure 2:**
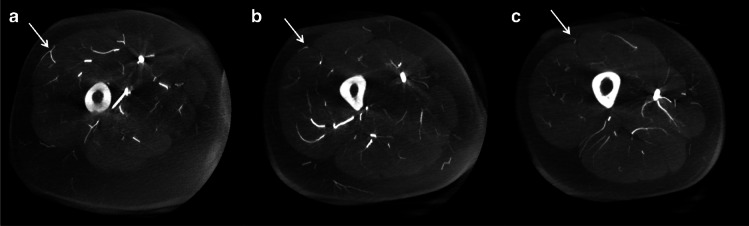
Flat-Panel Computed Tomography (FPCT) Imaging of a thigh for ALT flap perforator mapping. White arrow points at the point of fascial emergence (**a**–**c**).

The examination is performed as part of the digital subtraction angiography (DSA) of the respective extremity. All FPCT examinations were performed on a mono-planar flat-detector angiography system (Axiom Artis zee, Siemens Healthcare AG, Forchheim, Germany) after establishing a 4 French sheath in the common femoral artery on the donor site. 3D acquisition of the donor thigh was performed by manually injecting 10 mL of a water-soluble, non-ionic iodine contrast medium through the sheath (flow rate: 5 mL/s; Imeron 300 mg/mL; Bracco GmbH, Konstanz, Germany). Postprocessing of all obtained data was performed using the standard software of the manufacturer on a dedicated workstation directly linked to the angiography system (Syngo 3D, Siemens Healthcare AG, Forchheim, Germany). Multiplanar reconstructions were processed in axial, coronar and sagittal planes using 1 mm slice thickness and 1 mm spacing (increment) between images. For detection of suitable perforators, windowing of the datasets was performed individually in each patient to suit the reviewers’ preference. In case of finding a suitable perforator, the location of each point of passage through the superficial fascia was marked using the above named workstation (Fig. 2). Next, we automatically calculated an orthogonal distance between the cutis surface and the emerging point through the fascia. The virtual point of entry on the cutis surface was marked with a pen using the integrated function “Needle Guidance” (syngo Needle Guidance, Siemens Healthcare AG, Forchheim, Germany), which projects the intended orthogonal angulation of a desired pathway using a laser crosshair integrated in the angiography detector. With the help of this tool, we were able to mark each detected perforator directly on the cutis surface. All FPCT examinations were performed by the same independent interventional radiologist, who was blinded to the results of CCDS and TOF-MRA. The markings of the selected perforators according to the laser crosshair were performed by the study coordinator during the examination directly at the intervention table. This intends to minimize the risk of transfer errors due to patient movement as much as possible.

### Time-of-flight magnetic resonance angiography (TOF-MRA)

**Figure 3:**
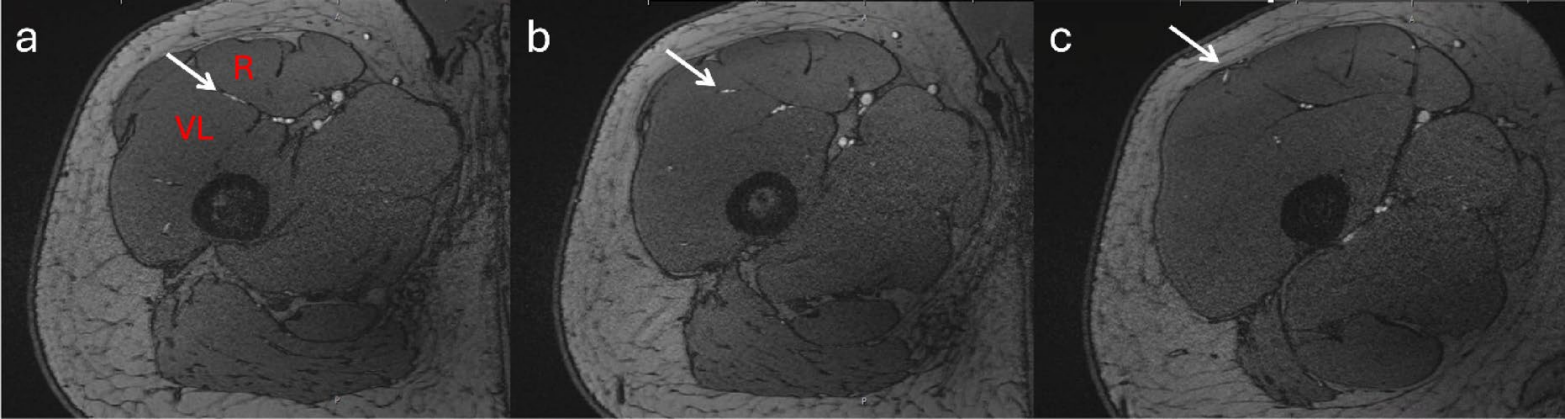
Time-of-Flight Magnetic Resonance Angiography (3 Tesla) of a thigh for ALT flap perforator mapping. White arrows point at the perforator throughout its course. (**a)** Shows the perforator running in the septum between the rectus femoris muscle (R) and the vastus lateralis muscle (VL). (**3)** Shows its further course in the vastus lateralis muscle. (**3)** Shows the perforator at the point where it pierces the fascia entering the subcutaneous fat.

All MRA examinations were performed on a clinically approved 3 Tesla MRI system (MAGNETOM Terra, Siemens Healthcare GmbH, Erlangen, Germany) using a 3-dimensional Time-of-Flight (TOF) sequence without venous saturation. The distance between the anterior superior iliac spine and the superior lateral border of the patella was divided into thirds for the markings. As reference points, two fish oil capsules were placed on the donor thigh, one at the transition between the upper and middle third and one between the middle and lower third. Both thighs were sequentially placed in a 18-channel body coil (Siemens Healthcare AG, Forchheim, Germany) covering most of the thigh in the direction of the z axis and centered in the middle third of the thigh markings. Suitable perforators for a planned ALT flap are expected in this area^29–31^. Correct positioning of the thigh was confirmed by the initial localizer, and adjusted if needed. TOF angiography was acquired with the following parameters: echo time, 3.69 ms; repetition time, 10 ms; voxel size, 0.3 × 0.3 × 0.8 mm; flip angle, 12 degrees (constant flip angle mode); FOV read 350 mm (FOV Phase 45%); and acquisition time, 12:27 min per leg. After completion of the TOF-MRA, potentially suitable perforators, their fascial emergence point and course were always determined by the same experienced radiologist without him knowing the results of CCDS and FPCT (Fig. 3). The coordinates of the fascial emergence points projected onto the skin surface and the course of each perforator were then passed on to the study coordinator, who carried out the markings on each patient. This imaging technique does not require any contrast agent application.

### Perforator assessment

In all three imaging modalities, the perforator localization is determined at the fascial emergence point and marked in color (blue: CCDS; black: FPCT; green: TOF-MRA) at the skin surface of the donor thigh using a permanent marker. In addition, the subfascial perforator course is determined and classified as follows:

1. Purely Septal
2. Mixed (mostly septal/little muscular)
3. Mixed (mostly muscular/little septal)
4. Purely Muscular

**Figure 4:**
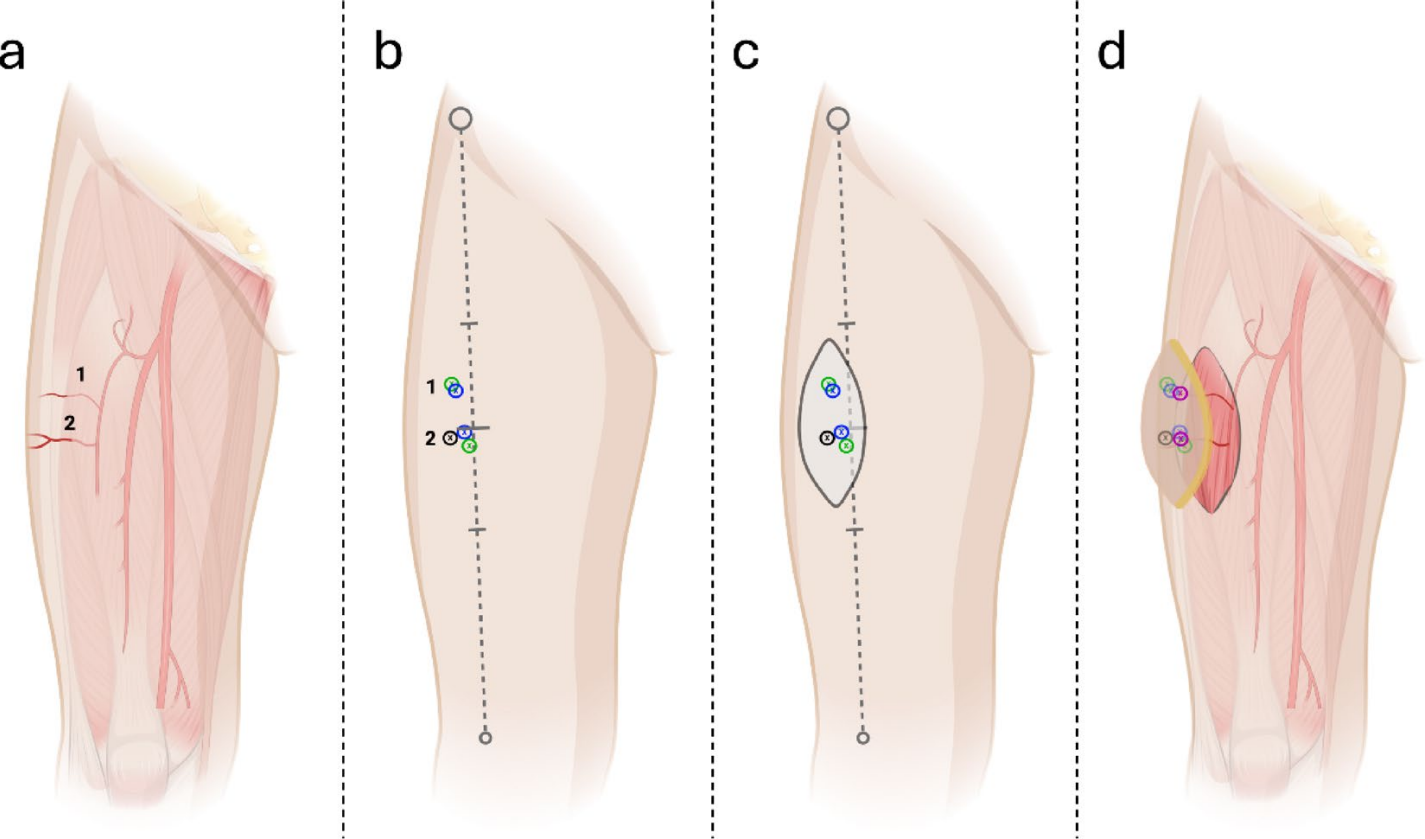
Exemplary graphical representation of the perforator marking process. (**a)** Shows two potential perforators with exit from the descending branch of the lateral circumflex femoral artery for ALT flap surgery. (**b)** Shows the marked fascial emergence point of both perforators using Color-Coded Duplex Sonography (blue), Flat-Panel Computed Tomography (black) and Time-of-Flight Magnetic Resonance Angiography (green). The black dotted line indicates the line connecting both reference points (anterior superior iliac spine and superolateral border of the patella) for ALT flap surgery. (**c)** Shows the ALT flap resection figure corresponding to the marked perforators and the reference line. (**d)** Shows the elevated ALT flap with the real fascial emergence point intraoperatively visualized and then transferred perpendicularly to the skin surface of the flap (purple markings). (Created with BioRender.com, accessed on the 7th of April 2025).

**Figure 5:**
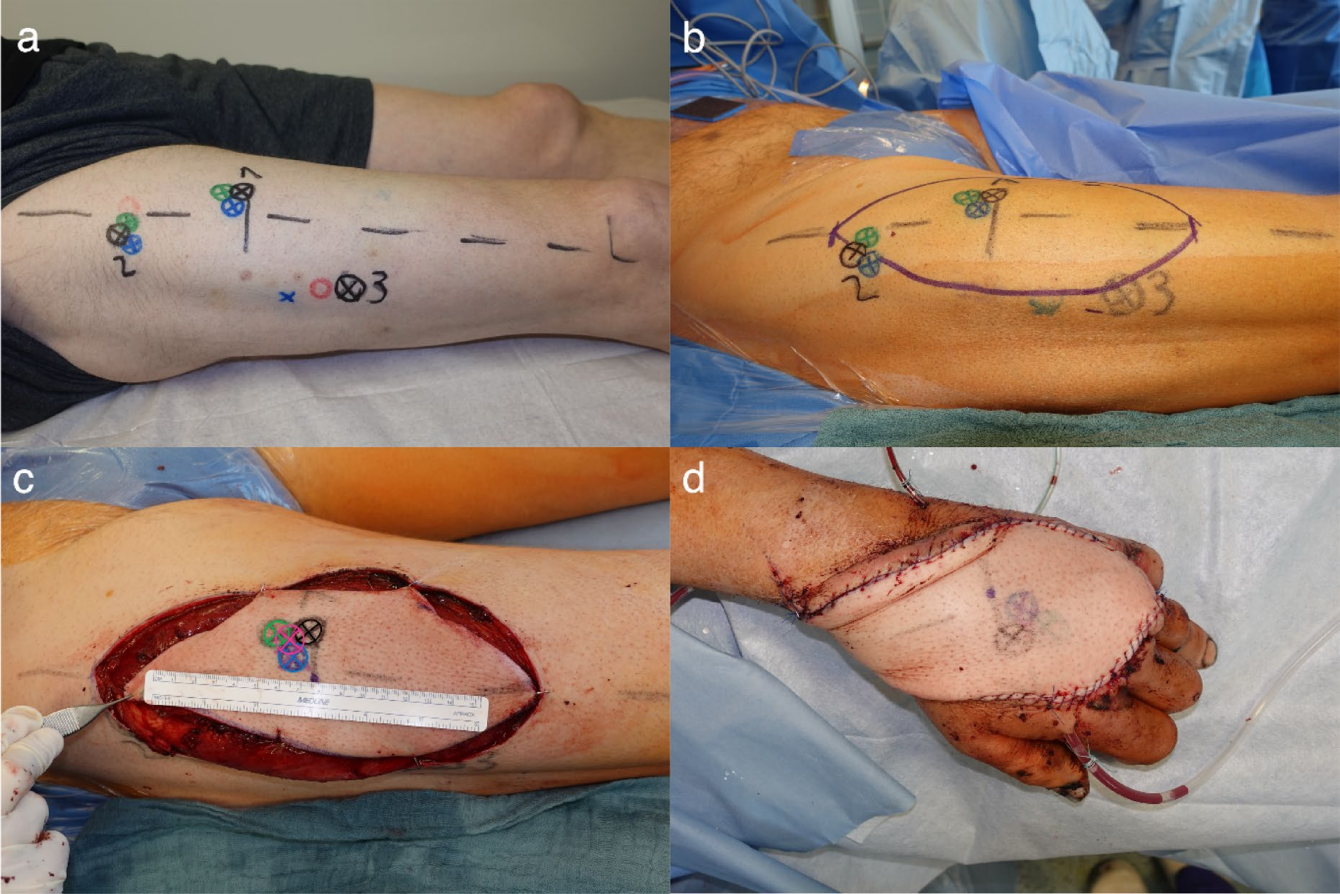
Clinical image series illustrating the individual steps of perioperative perforator marking and intraoperative verification. The color coding is defined as follows: blue: CCDS; black: FPCT; green: TOF-MRA; pink: intraoperative surgical verification. (**a)** Shows a total of three preoperatively detected perforators. The red markings show the former reference points of the fish oil capsules for the TOF-MRA. (**b)** Shows an image of the right thigh at the OR table with the flap design marked immediately prior to surgery. Of the marked perforators only a single one is situated within the boundaries of the flap design. (**c)** Shows an intraoperative view of the ALT flap displaying the markings from all three imaging modalities (CCDS, FPCT and TOF-MRA) as well as the actual intraoperative marking of the perforator’s fascial emergence point (pink marking). (**d)** Shows the vital ALT flap inset at the defect location on the left hand after completion of the microvascular anastomosis.

Intraoperative comparison of all three imaging modalities was performed with the actual fascial emergence point and perforator course. The real fascial emergence point was identified, projected perpendicularly and marked on the skin surface intraoperatively (Fig. 4). A standardized photograph including a measuring tape was taken (Fig. 5). After pedicle dissection, the surgeon documented the actual perforator course (1–4) and reported them to the study coordinator. Detection rate and accuracy of the fascial emergence point from each imaging modality were assessed by the study coordinator using the intraoperative photographs analyzed in Microsoft PowerPoint Version 16.96.1 (Microsoft Corporation, Redmond, Washington USA). Accuracy of perforator markings was defined as the distance between the marked and the actual fascial emergence point: “very close” (< 1 cm), “close” (1–2 cm) and “far” (> 2 cm)^22^.

### Statistical analysis

Descriptive analyses were applied to characterize flap and patient characteristics. Independence between two categorical variables was assessed using the Chi-squared, Fisher’s Exact or the Freeman-Halton test depending on table size and sample distribution. Post hoc analysis of combinations of categories was based on the square of the standardised residuals^32,33^. Statistical analyses were performed using GraphPad Prism (GraphPad Software, Inc., California, USA).

## Results

### Patient demographics

A total of 17 perforators from ten consecutive patients (one female, nine male) undergoing free ALT flap were included in this prospective study. Detailed patient characteristics are presented in Table 1. Of the 17 perforators identified within the flap design, 15 perforators were included in the flap pedicle (Table 1). CCDS was performed in all patients. FPCT was omitted in one case since contrast-enhanced vascular imaging of the defect area had already been performed recently and repeating contrast-enhanced imaging was deemed inadvisable. In two cases, TOF-MRA could not be performed due to metallic implants and in one case the examination had to be aborted due to claustrophobia (Table 2). The flap survival rate was 100% and no complications occurred during any image acquisition.

**Table 1:** Flap and patient characteristics.

***Flap Characteristics (n (%))***	
Total Number of Perforators	17 (100%)
Number of Perforators used for ALT Flap	15 (88.2%)
**Perforator Course**	15
Septal	2 (13%)
Mixed (mostly septal/little muscular)	1 (7%)
Mixed (mostly muscular/little septal)	6 (40%)
Muscular	6 (40%)
**Number of Flaps**	10
Survival	10 (100%)
Failure	0 (0%)
***Patient Characteristics (n (%))***	
**Gender Distribution**	
Female	1 (10%)
Male	9 (90%)
Mean Age ± SD (years)	50.1 ± 21.9
**Defect Location**	
Lower Leg	4 (40%)
Hand	5 (50%)
Upper Arm	1 (10%)
**Wound Etiology**	
Infection	3 (30%)
Trauma	6 (60%)
Tumor	1 (10%)

*ALT* anterolateral thigh, *SD* standard deviation.

**Table 2:** Imaging Modalities used for each patient.

Patient No	Imaging Modalities		
	CCDS	FPCT	TOF-MRA
1	x	x	x
2	x	x	-
3	x	x	x
4	x	x	-
5	x	x	x
6	x	x	x
7	x	x	x
8	x	x	-
9	x	-	x
10	x	x	x

*CCDS* Color-Coded Duplex Sonography, *FPCT* Flat-Panel Computed Tomography, *TOF-MRA* Time-of-Flight Magnetic Resonance Angiography.

### Perforator mapping

**Figure 6:**
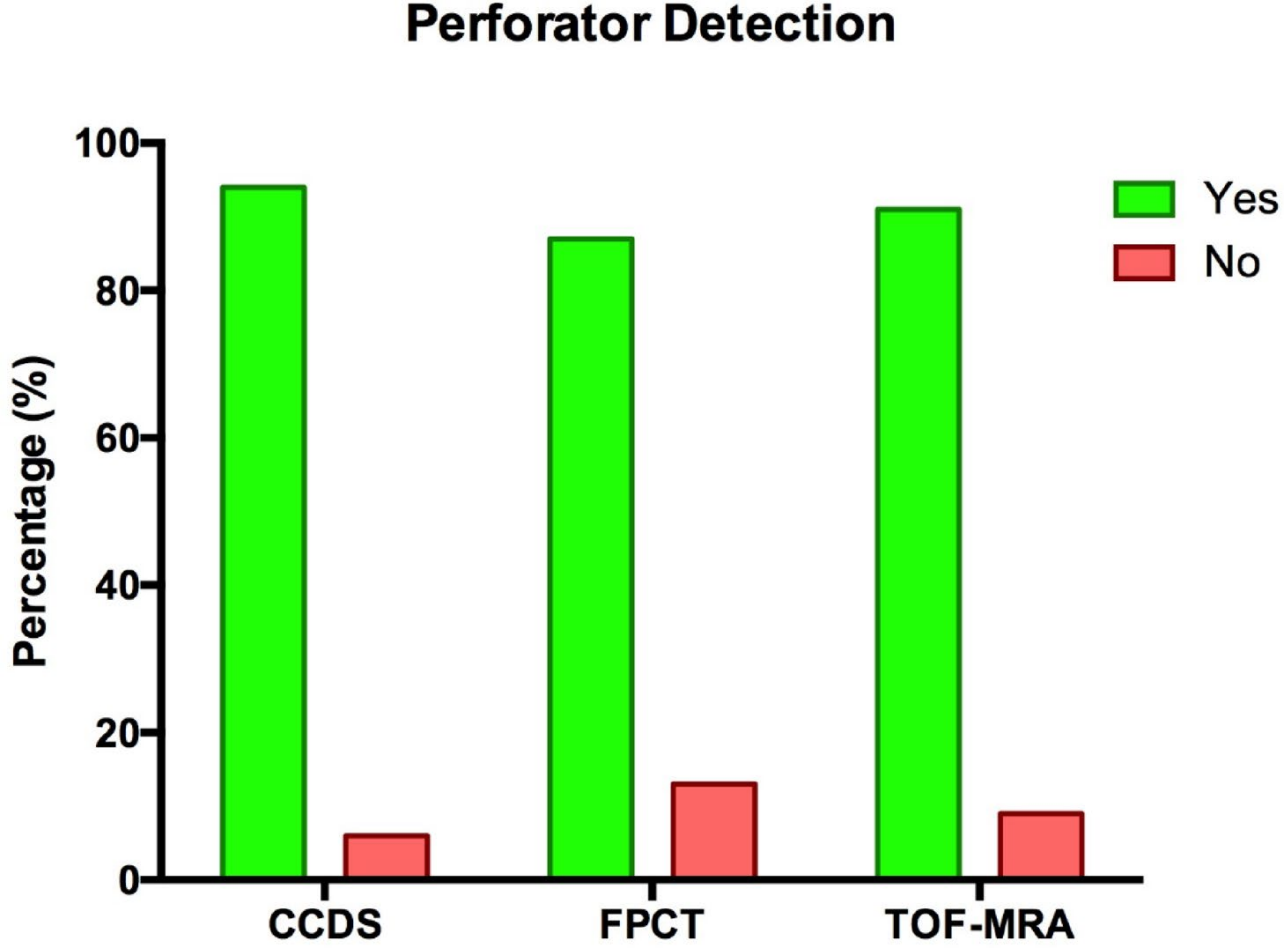
Perforator Detection Rate. Abbreviations: CCDS = Color-Coded Duplex Sonography; FPCT = Flat-Panel Computed Tomography; TOF-MRA: Time-of-Flight Magnetic Resonance Angiography.

**Figure 7:**
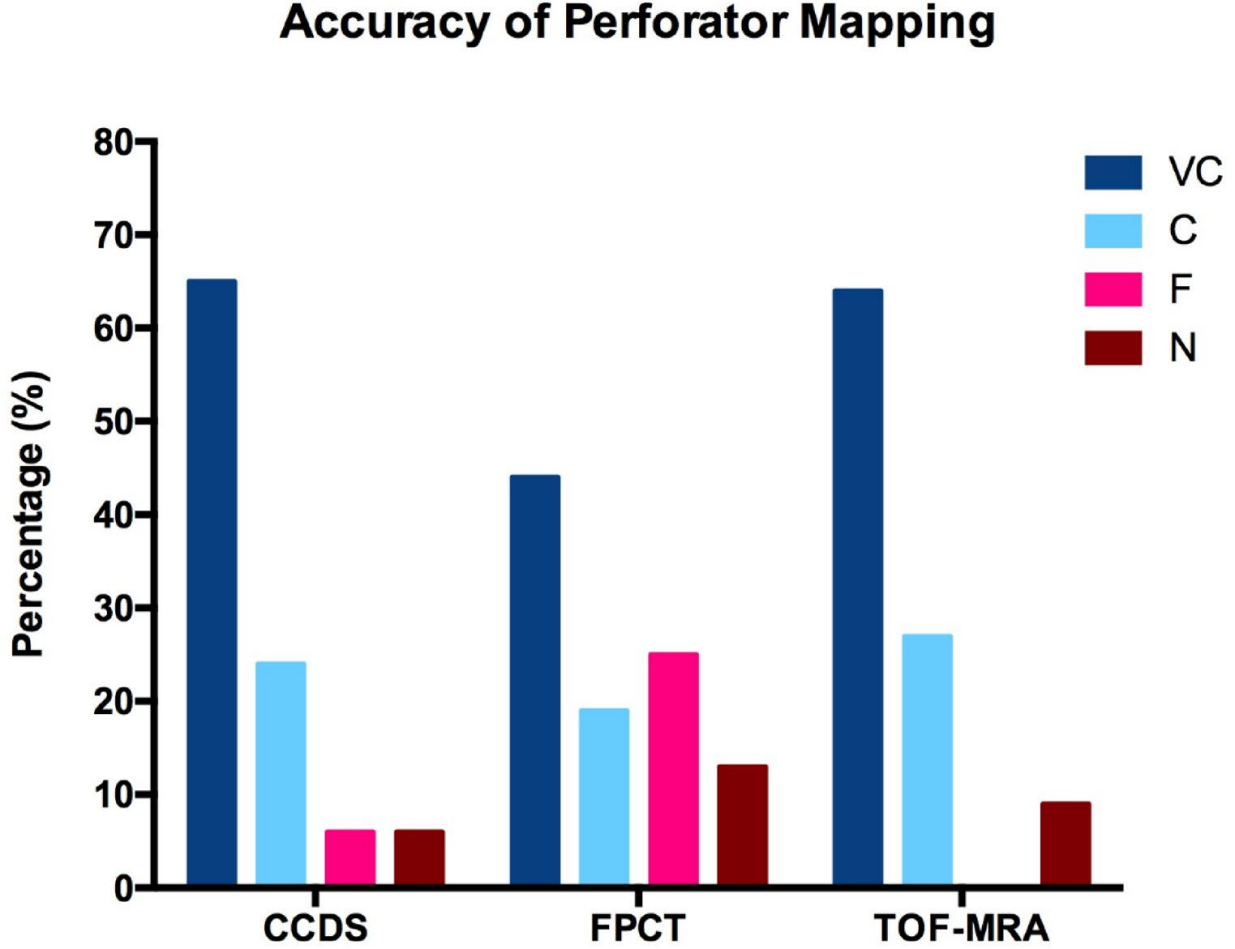
Accuracy of Perforator Mapping compared to intraoperative Perforator Position at the Fascial Emergence Point. Abbreviations: CCDS = Color-Coded Duplex Sonography; FPCT = Flat-Panel Computed Tomography; TOF-MRA: Time-of-Flight Magnetic Resonance Angiography; VC = Very Close (< 1 cm Radius); C = Close (1–2 cm Radius); F = Far (> 2 cm Radius); N = Not Detected.

**Figure 8:**
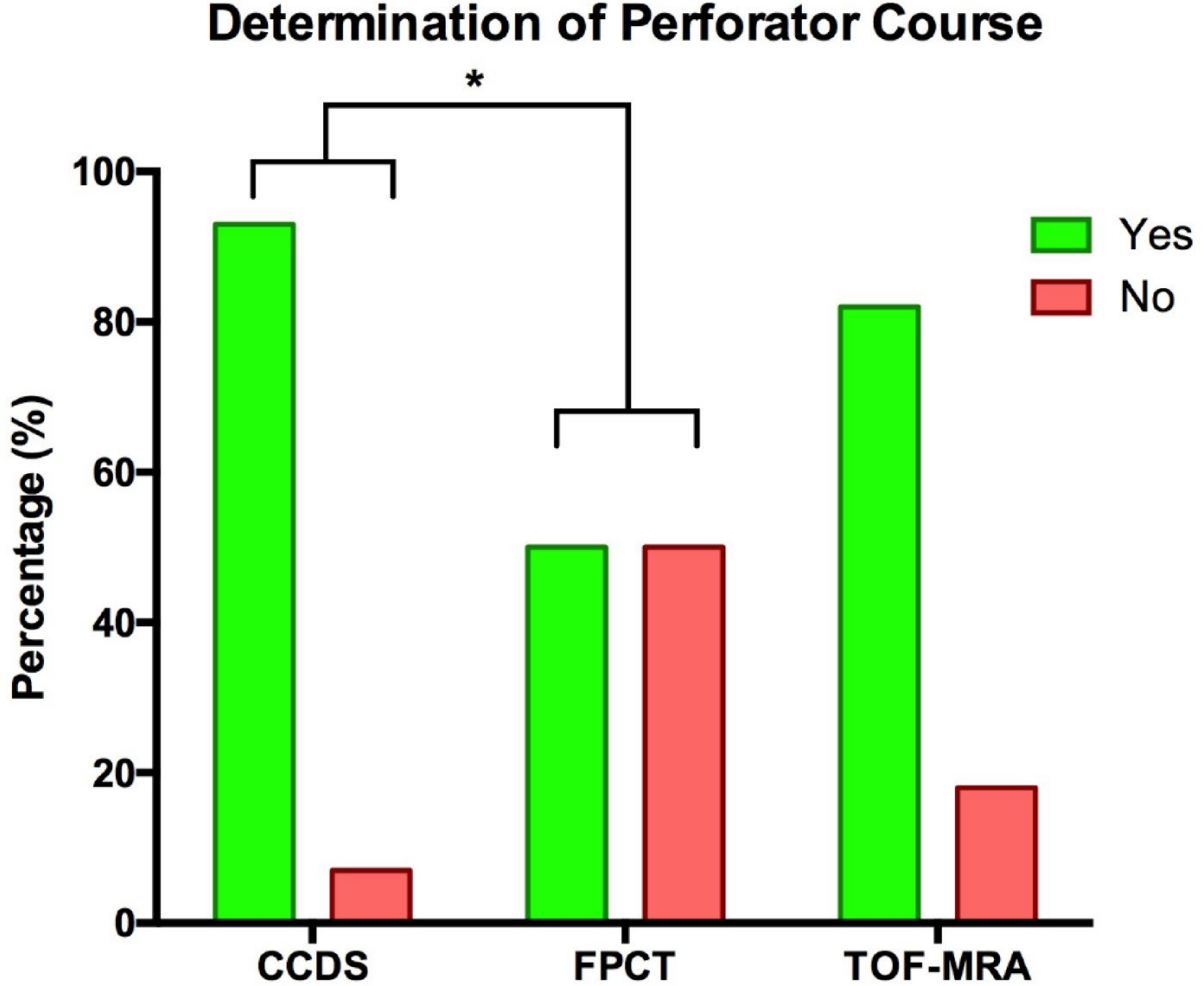
Determination of the Perforator Course (Septal/Mixed/Muscular). Abbreviations: CCDS = Color-Coded Duplex Sonography; FPCT = Flat-Panel Computed Tomography; TOF-MRA: Time-of-Flight Magnetic Resonance Angiography.

All preoperatively detected perforators identified by the imaging modalities were located within the planned flap design and were subsequently verified intraoperatively (Table 3). Perforators located outside the planned anterolateral thigh (ALT) flap design were excluded prior to skin marking and were therefore not considered in the analysis of this study. The detection rate of perforators used for ALT free flap transfer was highest with the CCDS at 94% (95% CI 71.3–99.9), which is consistent with the sensitivity of this imaging modality. Since every perforator detected with the ultrasound was intraoperatively present, the positive predictive value was 100%. The sensitivity was slightly lower for TOF-MRA at 91% (95% CI 58.7–99.8) and for FPCT at 87% (95% CI 61.7–98.4) (Fig. 6). Given the small sample size, formal statistical comparison using the Freeman-Halton test did not indicate a significant difference between modalities (*p* = 0.82). The positive predictive value of FPCT and TOF-MRA with regard to perforator detection was 100%. The accuracy of marked perforators showed that 65% of the perforators marked using CCDS were within 1 cm of the actual perforator (“very close”), compared with 64% for TOF-MRA and 44% for FPCT. The proportion of perforator markings within a 1–2 cm radius (“close”) was comparable across modalities (Fig. 7). However, FPCT showed a higher proportion of markings outside the 2 cm radius (“far”) (25%) compared with CCDS (6%) and TOF-MRA (0%). Overall, formal statistical testing did not demonstrate a significant difference in perforator marking accuracy between modalities (*p* = 0.31). The course of the perforator before fascial emergence is a relevant information for the surgeon preoperatively. In total, 2 of the 15 included perforators showed a purely septal course (13%), whereas six perforators had a purely muscular course (40%). The remaining seven perforators ran through both the septum and the musculature, six (40%) of which ran largely through the musculature and one (7%) of which was mostly septal. The CCDS showed the highest observed accuracy for preoperative prediction of the course of the perforator (93%; 95% CI 68.1–99.8) followed by TOF-MRA (82%; 95% CI 48.2–97.7) and FPCT (50%; 95% CI 23.0–77.0). An overall comparison did not demonstrate statistically significant differences between the three imaging methods in this respect. Nevertheless, a post-hoc pairwise comparison suggested a difference between CCDS and FPCT (*p* = 0.033) (Fig. 8). Given the small samples size this finding should be interpreted as exploratory.

**Table 3:** Overview of perforator detection and surgical verification by imaging modality.

Imaging Modalities	Examined Patients (n)	Preoperatively Detected Perforators (n)	Perforators Intraoperatively Verified in Flap Design (n)	Fully Dissected and Included Perforators (n)	False Negatives (n)	Unverified Perforator Markings
CCDS	10	16	17	15	1	0
FPCT	9	14	16	14	2	0
TOF-MRA	7	10	11	11	1	0

*CCDS* Color-Coded Duplex Sonography, *FPCT* Flat-Panel Computed Tomography, *TOF-MRA* Time-Of-Flight Magnetic Resonance Angiography.

## Discussion

Detailed preoperative knowledge of the number, course and caliber of perforators is essential for successful perforator flap surgery^19–21^. A survey among plastic surgeons revealed that 77% of respondents perform preoperative perforator mapping^34^. Despite its limited sensitivity and specificity, the handheld Doppler remains the most commonly used device. Although dependent on the device and probe frequency, the penetration depth can be up to 2 cm which theoretically carries the risk of detecting the axial vessel rather than the perforator itself, resulting in potential inaccuracies^22,35,36^. CCDS was used by 42% of surgeons, while CTA and MRA were only used by 7% and 8% of surgeons respectively^34^. CCDS has been shown to provide accurate and non-invasive visualization of the vascular anatomy with resolution in the millimeter range. Sensitivities up to 95%, specificities up to 100% and positive predictive values up to 97% have been reported for perforator detection in ALT flap surgery^31,37,38^. The present study compared CCDS, FPCT and non-contrast TOF-MRA regarding their detection rate, accuracy and visualization of the perforator course in patients undergoing free ALT flap surgery. CCDS demonstrated a sensitivity of 94% and a positive predictive value of 100% for the detection of perforators, confirming prior findings^37,38^. In terms of accuracy, CCDS achieved “close” or “very close” correlation with intraoperative position of perforators in nearly 90% of cases. Previous studies have demonstrated the feasibility of CT and Multidetector-Row CT (MDCT) angiography for preoperative perforator mapping^21,24^. However, the use of FPCT for perforator mapping in reconstructive surgery has not yet been described. FPCT integrated into angiographic C-arm systems enables distortion-free, high-resolution, CT-like imaging^27^. Originally developed for neurointerventional radiology, FPCT is increasingly applied for vascular, spinal and musculoskeletal imaging purposes^39–41^. In this study, FPCT showed a high sensitivity (87%) and positive predictive value (100%) for perforator detection. However, it has significant disadvantages such as radiation exposure and the intraarterial administration of contrast medium. Further, FPCT is heavily dependent on patient immobility during the examination. Any movement of the thigh between image acquisition and perforator marking which usually takes several minutes between acquisition and first results can cause substantial deviations leading to inaccurate perforator markings. In our study, this issue resulted in 25% of FPCT markings being classified as “far” (> 2 cm radius from the actual fascial emergence point) compared to only 6% with CCDS. In one patient, thigh movement during examination led to substantially inaccurate perforator markings. This highlights that the diagnostic effectiveness of FPCT strongly depends on strict immobilization during the procedure. TOF-MRA at 3 T magnetic field strength demonstrated a sensitivity of 91%, a positive predictive value of 100% and in 91% of cases a localization accuracy within a 2 cm radius to the true perforator position, similar to CCDS results. TOF-MRA offers key advantages such as minimal examiner dependency, no ionizing radiation and no requirement for contrast agents. Another key advantage is its ability to measure the thickness of subcutaneous fat around the selected perforator without the compression artifacts associated with CCDS. This enables perforator selection based on flap thickness and helps estimate the required pedicle length, both of which are important aspects for the reconstruction of complex defects such as those involving the oral cavity. A study by Wiesmueller et al. confirmed the feasibility of TOF-MRA for identifying peroneal perforators using 7 Tesla MRI^28^. However, 7 Tesla ultra-high field MRI systems are mainly limited to major centers, limiting its widespread clinical application. The present study used a 3 Tesla MRI system which makes it a more accessible and reproducible option. However, MRI technology may have inherent risks due to its strong magnetic field such as movement or heating of ferromagnetic material. In our study, two patients were excluded from TOF-MRA due to metallic fragments or external fixators. One examination was aborted due to claustrophobia. This highlights the need for alternative modalities such as CCDS or CT angiography technologies such as FPCT in patients contraindicated for TOF-MRA. CCDS also has relevant limitations including its strong dependency on examiner expertise requiring dedicated ultrasound training and substantial anatomical knowledge^17,37^. In addition, the availability of high-frequency ultrasound systems, such as the 20 MHz probe used in this study, is limited outside specialized tertiary hospitals. However, both FPCT and TOF-MRA are likewise limited by the requirement for radiologists experienced in perforator mapping interpretation as well as by their relatively high costs and resource demands. The availability of both of these advanced imaging modalities may vary depending on the financial resources of individual healthcare systems and may be particularly limited outside tertiary referral centers. Visualization of the entire perforator course from its origin to its fascial emergence point is another crucial aspect of ALT flap planning. According to the literature, 62.5–80% of perforators follow an intramuscular course which requires meticulous dissection increasing operative-time and complexity^14,37^. Some authors even postulate choosing the contralateral thigh or even refrain from an ALT flap entirely in cases with a long intramuscular course^22^. This highlights the necessity of identifying and understanding the perforator course in as much detail as possible. Therefore, the perforator course was divided into four categories (see Methods section). CCDS achieved the highest accuracy (93%) in predicting the perforator course, consistent with previous studies^22,37^. Similar results were obtained with TOF-MRA with an accuracy of 82%. However, image quality strongly depended on an optimal coil placement. The more central the coil was positioned over the target perforator, the better the visualization. FPCT showed significantly lower accuracy in predicting the perforator course than CCDS, due to its limited soft-tissue contrast resolution. The limitations of this study include its relatively small sample size, possible verification bias due to incomplete imaging across all patients, non-independence of clustered observations and selection bias due to the single-center study design. Since only perforators within the flap design were surgically verified, the reported positive predictive value and sensitivity reflect the performance only within the described clinical surgical setting. Therefore, our results should be considered exploratory estimates. Furthermore, observer bias due to the absence of interobserver reliability assessment as well as the lack of correlation with postoperative outcomes, operative time, dissection difficulty and intraoperative changes in flap design are limitations of this study. A methodological limitation of this study is the potential time interval between imaging and perforator markings specifically in TOF-MRA which makes it susceptible to transfer errors due to skin displacement or patient movement.

## Conclusion

This study highlights the value of CCDS as an effective imaging modality for preoperative perforator mapping of ALT flaps. This may reduce its reliability in centers with low ALT flap volumes. Our results indicate that TOF-MRA at 3 Tesla, performed without contrast agents, offers a reliable and less examiner-dependent alternative. However, general MRI contraindications, such as metallic implants or claustrophobia, resulted in a notably high exclusion rate of 30% and may be regarded as a main obstacle. FPCT is a suitable option especially in cases where vascular imaging, such as digital subtraction angiography, of the lower extremity is required anyway. While FPCT appeared to provide less accurate visualization of the perforator course within soft tissue than CCDS, its high spatial resolution allows reliable perforator detection and precise marking of the fascial emergence point. Patient immobilization is a crucial factor for reliable results of this otherwise robust technique. The relatively small sample size of this study precludes a definitive recommendation for either imaging modality. Nevertheless, it can be concluded that CCDS remains a highly accurate technique, particularly in high-volume centers with extensive sonographic expertise. In contrast, TOF-MRA, with its non-invasiveness and lower operator dependency, appears to be a suitable alternative for institutions with fewer ALT flap procedures. Despite limitations such as its invasiveness, exposure to radiation, the use of contrast dye and its sensitivity to patient movement, FPCT is considered a justified tool, particularly when contrast-enhanced vascular imaging of the respective extremity is required. Although FPCT and TOF-MRA are well-established in clinical routine for other reasons, they have yet to be widely adopted as tools in routine perforator mapping. This study highlights the feasibility and potential of advanced imaging technologies in perforator diagnostics to enhance reconstructive surgical planning. Considering the above mentioned study limitations, the findings should be interpreted as exploratory and hypothesis-generating rather than definitive.

## Supplementary Information

Below is the link to the electronic supplementary material.

Supplementary Material 1.

## Data Availability

The datasets used and analysed during the current study are available from the corresponding author on reasonable request.
